# Risk factors for complicated diverticulitis: systematic review and meta-analysis

**DOI:** 10.1007/s00384-017-2872-y

**Published:** 2017-08-10

**Authors:** H. E. Bolkenstein, B. J. M. van de Wall, E. C. J. Consten, I. A. M. J. Broeders, W. A. Draaisma

**Affiliations:** 1 Department of Surgery, Meander Medisch Centrum, 3813 TZ, Amersfoort, Netherlands; 2 Department of Surgery, Jeroen Bosch Ziekenhuis, Den Bosch, Netherlands

**Keywords:** Diverticulitis, Acute, Complicated, Risk factors, Score

## Abstract

**Purpose:**

The aim of this systematic review is to identify risk factors that can predict complicated diverticulitis. Uncomplicated diverticulitis is a self-limiting and mild disease, but 10% of patients with diverticulitis develop complications requiring further treatment. It is important to estimate the risk of developing complicated diverticulitis at an early stage to set the right treatment at initial presentation.

**Methods:**

Embase, MEDLINE, and Cochrane databases were searched for studies reporting on risk factors for complicated diverticulitis. Complicated diverticulitis was defined as Hinchey ≥Ib or severe diverticulitis according to the Ambrosetti criteria. Meta-analyses were performed when at least four studies reported on the outcome of interest. This study was conducted according to the PRISMA guidelines.

**Results:**

A total of 12 studies were included with a total of 4619 patients. Most were of reasonable quality. Only the risk factors “age” and “sex” were eligible for meta-analysis, but none showed a significant effect on the risk for complicated diverticulitis. There was reasonable quality of evidence suggesting that high C-reactive protein; white blood cell count; clinical signs including generalized abdominal pain, constipation and vomiting; steroid usage; a primary episode; and comorbidity are risk factors for complicated diverticulitis.

**Conclusion:**

Although high-level evidence is lacking, this study identified several risk factors associated with complicated diverticulitis. Individually, these risk factors have little value in predicting the course of diverticulitis. The authors propose a prognostic model combining these risk factors which might be the next step to aid the physician in predicting the course of diverticulitis and setting the right treatment at initial presentation.

## Introduction

In the Netherlands, approximately 22,000 patients per year are referred to secondary care with diverticulitis [1, 2]. Ten percent of these patients will develop complications such as abscess or perforation and require further treatment in the form of close observation, antibiotics, percutaneous drainage, or surgery. Uncomplicated diverticulitis is however a self-limiting and relatively mild disease [3, 4]. Recent literature has indicated that the outpatient treatment of uncomplicated diverticulitis is safe and effective [5, 6]. This implies that uncomplicated diverticulitis can be safely treated in primary care. The National Guideline for general practitioners (NHG standard) considers diverticulitis a clinical diagnosis based on the following signs: the development of persistent sharp, stabbing pain in the lower left abdomen within a couple of days and pressure or rebound tenderness only in the lower left abdomen. CRP level above 20 mg/L and body temperature > 38.0 °C could support the diagnosis. Ultrasound or CT scan is deemed unnecessary in the primary care setting when the abovementioned symptoms are present. The NHG standard advises only to refer patients with a suspicion of complicated diverticulitis to secondary care [1]. However, a considerable amount of patients with uncomplicated diverticulitis is still referred to secondary care, resulting in unnecessary diagnostics (ultrasound, CT scan) and treatment (antibiotics, hospital admittance). To reduce the annual health-care costs of diverticulitis and improve diverticulitis care, these unnecessary referrals should be reduced. Such a strategy would demand a proper prognostic tool to help estimate the risk of developing complicated diverticulitis, since this estimation will influence the course of action of the treating physician. If the treating physician can more accurately predict the course of the disease after setting the diagnosis, he will feel more comfortable to treat patients at home. To this day, there are no prognostic models that can predict the severity of diverticulitis. More evidence on risk factors for complicated diverticulitis is needed to establish such a model and aid the treating physician in predicting the course of diverticulitis and setting the right treatment at initial presentation. Therefore, a systematic review and meta-analysis were performed to identify risk factors for complicated diverticulitis.

## Material and methods

### Search strategy

This systematic review was conducted according to the Preferred Reporting Items for Systematic Reviews and Meta-analysis (PRISMA) guidelines and was executed in May 2016 [7]. The databases PubMed, Embase, and Cochrane library were searched using synonyms for domain (diverticulitis), determinant (risk factors), and outcome (complicated diverticulitis). The used search terms are listed in appendix 1. The search results were filtered for doubles, and the remaining articles were screened for title and abstract. All studies that did not report on the domain (diverticulitis) and outcome (complicated diverticulitis) were excluded. All non-English publications and studies performed before 1990 were also excluded.

The remaining articles were read for full text. Only studies comparing patients with uncomplicated to complicated diverticulitis were included in this review. Case-reports, expert-opinions, reviews, and studies on right-sided diverticulitis were excluded. The references of all selected studies were hand-searched for other relevant studies. Ambiguities were resolved by consultation with the senior authors.

### Data extraction

Data regarding study characteristics and all relevant risk factors were extracted. A risk factor for complicated diverticulitis encompassed all patient characteristics (medical history, age, gender, body mass index, race), clinical signs (pain, nausea, vomiting, rectal bleeding, diarrhea, constipation), physical signs (guarding, palpable mass, signs of bowel obstruction), vital signs (body temperature, heart rate, blood pressure, respiratory rate), and laboratory parameters (C-reactive protein (CRP) white blood cell (WBC) count, sodium).

Uncomplicated diverticulitis was defined as Hinchey Ia diverticulitis or “mild diverticulitis” according to the Ambrosetti classification. Complicated diverticulitis was defined as Hinchey ≥Ib or severe diverticulitis according to the Ambrosetti criteria (see appendix 2 and 3).

### Critical appraisal

All selected articles were critically appraised by H. Bolkenstein. Cross-sectional studies were assessed on relevance and quality using the cross-sectional appraisal tool from wordpress.com [8]. Longitudinal studies were assessed using the Scottish Intercollegiate Guidelines Network (SIGN) Methodology Checklist http://www.sign.ac.uk/pdf/sign50annexc.pdf. Studies that were considered poorly were excluded to ensure the quality of the systematic review and minimize risk of confounding and bias. Ambiguities were resolved by consultation with the senior authors.

### Data analysis

Review Manager (RevMan) software version 5.1 was used for the meta-analysis. Pooling of data was only performed of studies reporting mean and standard deviation and when at least four studies reported on the outcome of interest. The remaining outcomes were described qualitatively. Pooled risk ratios (RRs) comparing uncomplicated diverticulitis to complicated diverticulitis were calculated using a random effects model allowing for variation beyond chance in estimates across studies. The I2 statistic was used to quantify the amount of heterogeneity. To obtain insight on the absolute cumulative risk of determinants, the authors used the average risk across studies.

## Results

### Search and critical appraisal

**Fig. 1:**
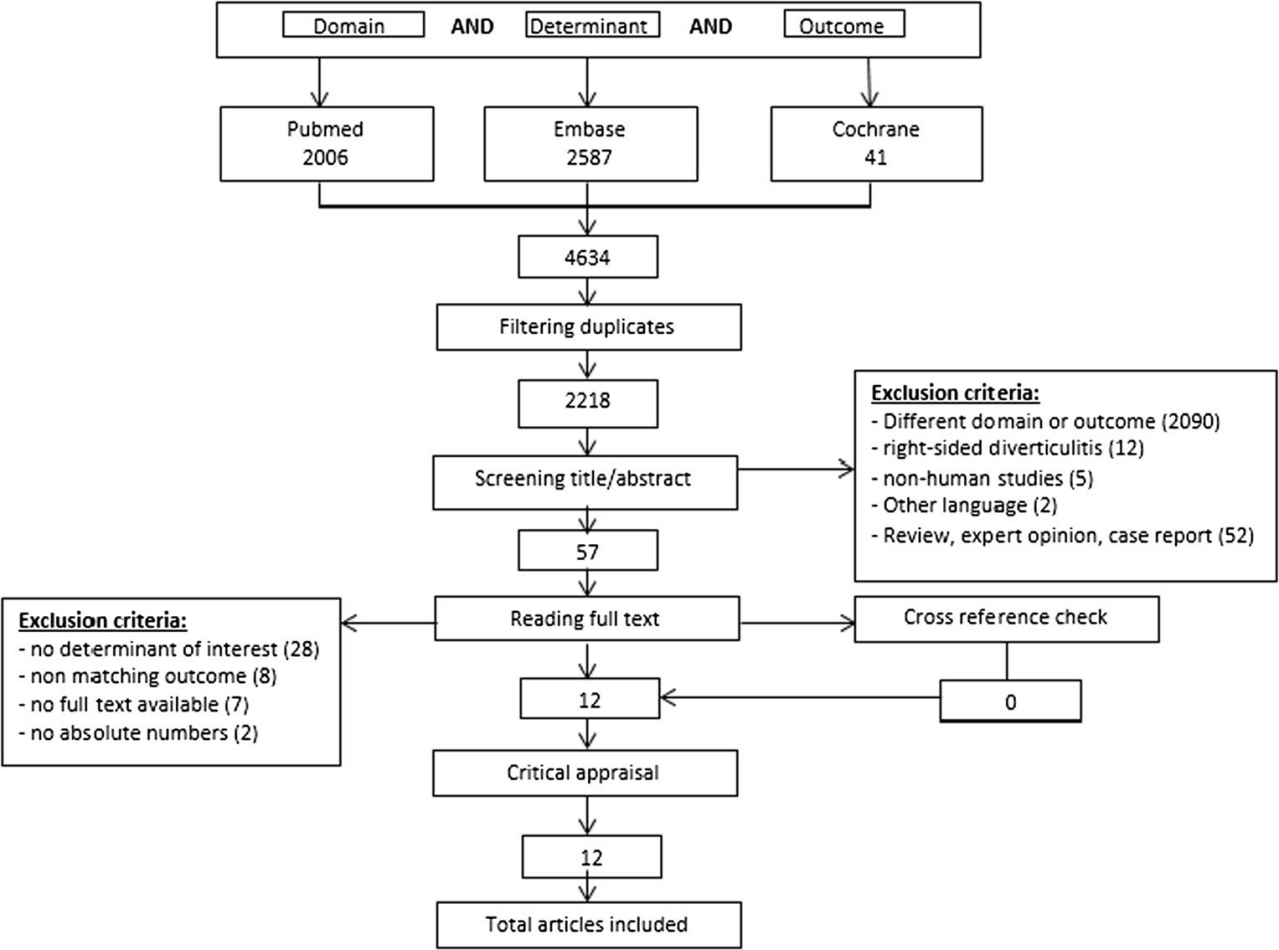
Search results

Search results and study selection are described in Fig. 1. A total of twelve articles were critically appraised [9–20]. Most studies were rated as reasonable to good quality based on the cross-sectional appraisal tool from wordpress.com and the SIGN Methodology Checklist [8] http://www.sign.ac.uk/pdf/sign50annexc.pdf. Studies were mostly downgraded due to lack of control of possible confounders. None of the studies were graded high quality since the studies were of retrospective design and/or had small patient numbers and/or poor presentation of results. The results of the critical appraisal are depicted in Table 1.

**Table 1:** Study characteristics

Study	Design	Quality assessment		Number of patients	Men (%)	Age (years)	Study population	Definition complicated diverticulitis	UC	C
		SIGN checklist^a^	Cross-sectional appraisal tool^b^							
Cologne 2014	Retrospective cohort	+		1019	13.1	49.8	Radiologically confirmed diverticulitis, any episode	Hinchey >1A or “severe” diverticulitis according to Ambrosetti criteria	838	181
Faria 2011	Retrospective cohort	+/−		157	43.3	60	Radiologically or pathologically confirmed diverticulitis, any episode	Hinchey >1A	107	50
Hall 2010	Retrospective cohort	+/−		932	48	61	Radiologically confirmed diverticulitis, any episode	“Severe” diverticulitis according to Ambrosetti criteria	806	126
Hjern 2008	Retrospective cohort	+/−		234	NR	59	Radiologically or pathologically confirmed diverticulitis, any episode	“Severe” diverticulitis according to Ambrosetti criteria	212	22
Kotzampassakis 2010	Retrospective cohort	+/−		271	52.4	60	Radiologically confirmed diverticulitis, any episode	“Severe” diverticulitis according to Ambrosetti criteria	166	105
Longstreth 2012	Retrospective cross sectional		+	741	43.7	57.2	Radiologically confirmed diverticulitis, any episode	Hinchey >1A	649	92
Makela 2015	Retrospective cross sectional		+	350	42	60	Radiologically confirmed diverticulitis, first episode	Hinchey >1A	169	181
Nizri 2013	Retrospective cross sectional		+	295	NR	NR	Radiologically confirmed diverticulitis, any episode	Hinchey >1A	243	52
Pisanu 2013	Retrospective cohort		+/−	80	56.2	60	Radiologically confirmed diverticulitis, any episode	Hinchey >1A	30	50
Tursi 2008	Prospective cross sectional		+/−	50	42	63.6	Radiologically confirmed diverticulitis, any episode	Hinchey >1A	39	11
Van de Wall 2012	Retrospective Cross-sectional		+	426	NR	NR	Radiologically confirmed diverticulitis, any episode	Hinchey >1A	364	62
West 2003	Retrospective cohort	+/−		64	NR	45.5	Radiologically or pathologically confirmed diverticulitis	“Severe” diverticulitis according to Ambrosetti criteria	38	26

*UC* uncomplicated diverticulitis, *C* complicated diverticulitis, ++ high quality, + good, +/− reasonable, – poor

^a^Scottish Intercollegiate Guidelines Network (SIGN) Methodology Checklist

^b^Cross-sectional appraisal tool, https://reache.files.wordpress.com/2010/03/cross-sectional-appraisal-tool.pdf, based on; Guyatt GH, Sackett DL, Cook DJ, Users’ guides to the medical literature. II. How to use an article about therapy or prevention. JAMA 1993; 270 (21): 2598–2601 and JAMA 1994; 271 (1):59–63

### Baseline characteristics

Study characteristics of the included studies are shown in Table 1. Of the twelve included studies, four had a retrospective cross-sectional design [14–16, 19] and seven had a retrospective cohort design [9–13, 17, 20]. There was one prospective cross-sectional study [18]. The 12 included articles evaluated a total of 4619 patients with diverticulitis. In all studies, the diagnosis (complicated) diverticulitis was proven by computed tomography (CT) or pathological examination. A total of 3661 (79%) patients had uncomplicated diverticulitis and 958 (21%) had complicated diverticulitis.

### Main outcome—risk factors for complicated diverticulitis

#### Age

**Fig. 2:**
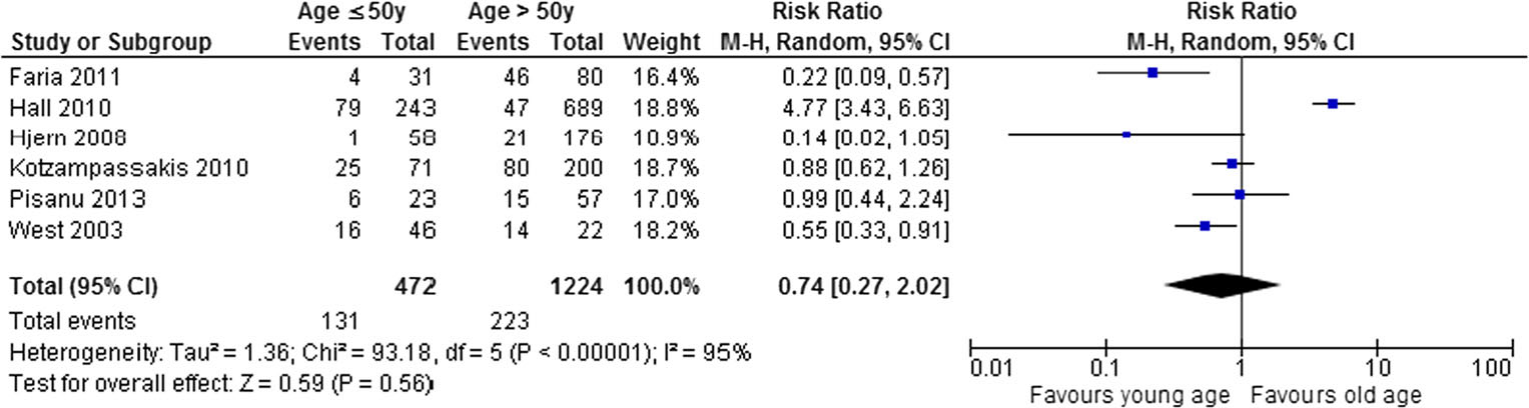
Meta-analysis. Age ≤ 50 and > 50 years

Ten studies reported on age as a risk factor for complicated diverticulitis [10–17, 19, 20]. A pooled data analysis was performed on studies that reported age as a dichotomous variable (older or younger than 50 years) [10–13, 17, 20]. The pooled analysis showed no significant difference. The pooled risk ratio was 0.74 (95% CI 0.27–2.02) in a random effects model (*I* ^2^ = 95%), as depicted in Fig. 2. Makela et al. reported the influence of age on the risk for complicated diverticulitis in three groups (< 50, 50–70, and > 70 years). They found a significant effect of old age (> 70 years) on the risk of complicated diverticulitis (*p* = 0.008) [15].

Pooling of studies that described age as a continuous variable (mean and standard deviation) was not possible due to the fact some studies did not report the required standard deviation of the mean age. There was no consensus among these studies on the effect of age on the severity of diverticulitis. Van de Wall et al. found that patients with complicated diverticulitis were of a significantly (*p* < 0.05) higher age (63.9 years) as compared with patients with an uncomplicated episode (57.1 years) [19]. Nizri et al. and Longstreth et al. however did not find a significant effect. They respectively found a mean age of 63 and 57.3 years in patients with an uncomplicated episode compared to 59.3 and 56.6 years in patients with complicated diverticulitis (*p* = 0.182 and 0.71, respectively) [14, 16].

#### Gender

**Fig. 3:**
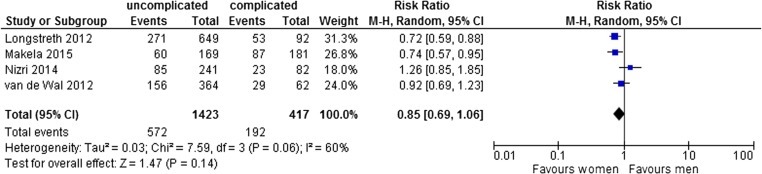
Meta-analysis. Sex (number of men)

Four studies reported on gender [14–16, 19]. Pooled analysis demonstrated no significant difference in risk for complicated diverticulitis. The pooled risk ratio was 0.85 (95% CI 0.69–1.06) in a random effects model (*I* ^2^ = 60%), as depicted in Fig. 3. The absolute risk of developing complicated diverticulitis varied from 9 to 46% in women with an estimated average of 21%. In men, the absolute risk of developing complicated diverticulitis varied from 16 to 59% with an estimated average of 25%.

#### History of previous attacks

Two studies reported on history of previous attacks as a risk factor for complicated diverticulitis [16, 19]. Nizri et al. found that a primary episode of diverticulitis was at greater risk to be accompanied by complications compared to recurrent episodes (RR 1.98, 95% CI 1.26–3.11) [16]. Van de Wall et al. did not find a significant effect of previous attacks on the severity of diverticulitis. Twelve percent of the patients who presented with uncomplicated diverticulitis had had previous attacks, compared to 14% of the patients presenting with complicated diverticulitis [19].

#### Clinical signs and physical examination

Three studies reported on clinical signs (such as nausea, vomiting, bloating) as risk factors for complicated diverticulitis [14, 18, 19]. Longstreth et al. found that significantly more patients with complicated diverticulitis had signs of constipation (OR 2.32, 95% CI 1.27–4.23). Furthermore, patients with complicated diverticulitis presented less frequently with localized pain in the lower left abdomen (OR 0.54, 95% CI 0.29–0.99). These patients had more generalized abdominal pain [14].

Van de Wall et al. found that patients with a complicated episode presented more frequently with vomiting (26 versus 11%) and diffuse abdominal pain (20 versus 9%) than patients with an uncomplicated episode [19].

Tursi et al. investigated the severity of symptoms in uncomplicated and complicated diverticulitis graded on a quantitative scale. They found that patients with complicated diverticulitis had more severe constipation, abdominal pain, and, when present, more severe rectal blood loss [18].

#### Body temperature

Body temperature at presentation was reported in three studies. Tursi et al. found that a temperature greater than 37 °C was associated with complicated diverticulitis. Almost all patients (9 out of 11) with complicated diverticulitis presented with a temperature greater than 37 °C while all the patients with uncomplicated diverticulitis (39 out of 39) had a temperature below 37 °C [18].

Longstreth et al. demonstrated that patients presenting with a temperature greater than 37.5 °C had a higher risk of having complicated diverticulitis (OR 2.13, 95% CI 1.27–3.57). Van de Wall reported on mean body temperature and did not find a significant effect. The mean temperature in patients with uncomplicated diverticulitis was 37.5 °C (36.2–38.9) and 37.6 °C (36.3–39.0) for complicated cases [14].

#### C-reactive protein

Four studies reported on CRP level as a risk factor for complicated diverticulitis [15, 16, 18, 20]. All studies found a significant effect of CRP level on the risk of complicated diverticulitis. The overall mean CRP among patients with uncomplicated diverticulitis was 68 mg/L with a range of 25 to 96 mg/L. This was 186 mg/L with a range of 134 to 224 mg/L among patients with complicated diverticulitis.

Three studies calculated the optimal threshold value of CRP level to distinguish uncomplicated from complicated diverticulitis. Makela et al. found an optimal cutoff point of 149.5 mg/L (sensitivity 65%, specificity 85%) [15]. The studies of Nizri et al. and van de Wall et al. found an optimal cutoff point of 90 mg/L (sensitivity 88%, specificity 75%) and 175 mg/L (sensitivity 61%, specificity 82%), respectively [16, 19].

#### White blood cell count

Four studies reported on this risk factor [14, 15, 18, 19]. Tursi et al. and van de Wall et al. reported on WBC as a continuous variable showing a significant effect of WBC on the risk of complicated diverticulitis. Average mean WBC count was 10.4 × 10^9^/L (range 8.7–12.0 × 10^9^/L) in uncomplicated diverticulitis and 14.4 × 10^9^/L (range 12.5–15.3 × 10^9^/L) in complicated diverticulitis [18, 19].

Two studies reported WBC as a dichotomous value [14, 15]. Makela et al. reported a sensitivity of 51% and specificity of 46% for a cutoff value of 10 × 10^9^/L (*p* = 0.672) [15]. Longstreth et al. found a sensitivity of 82% and specificity of 45% for a cutoff value of 11 × 10^9^/L (*p* = < 0.0001) [14].

#### Body mass index

Only one study reported on body mass index (BMI) as a risk factor for complicated diverticulitis. Longstreth et al. found no significant difference between patients with a BMI greater or smaller than 25 (OR 1.00 (CI 0.96–1.04) [14].

#### Comorbidity

One study reported on comorbidity and found that the group of patients with complicated diverticulitis consisted of patients with a higher American Society of Anesthesiologists (ASA) classification (ASA I 26%; ASA II 65%; ASA III 10%) compared to the group with uncomplicated diverticulitis (ASA I 41%; ASA II 51%; ASA III 8%) [19].

#### Diabetes mellitus

The effect of diabetes mellitus (DM) on the risk of complicated diverticulitis was reported in one retrospective cohort study. Approximately 16% of the patients without DM had complicated diverticulitis compared to 27% of the patients with DM (*p* < 0.003) [9].

#### Steroid use and immunosuppression

One study reported on the use of steroids. Patients with complicated diverticulitis more frequently used steroids compared to patients with uncomplicated diverticulitis (7.3 versus 3.3%; *p* = 0.015) [16].

## Discussion

### Summary of results

This systematic review and meta-analysis included 12 studies with a total of 4619 patients. Few studies were found that accurately described risk factors for complicated diverticulitis. Most of the studies were of retrospective design and did not account for confounders in their analyses. The evidence in the current literature for risk factors for complicated diverticulitis is therefore not strong. Considering the high incidence of this disease and the high impact on health, quality of life, and health-care costs, this topic deserves more attention. This systematic review found that CRP, WBC count, and clinical signs (constipation, generalized abdominal pain, and vomiting) are risk factors for complicated diverticulitis. Comorbidity, number of episodes, and steroid usage were suggested as possible risk factors, but evidence for these parameters was not very strong. Only the parameters “age” and “sex” were eligible for meta-analysis. None of these parameters showed a significant difference in the risk for complicated diverticulitis.

### Limitations of the study

There are some limitations of this study that should be taken into account when interpreting the results. In this systematic review, there was significant heterogeneity in the design and methods of the included studies. We accounted for this problem by using a random effects model which yields a more conservative estimate in case of heterogeneity. Additionally, we only included studies with the same definition of complicated diverticulitis, thus minimizing the heterogeneity in outcome encountered in previously published reviews.

Most risk factors were not eligible for meta-analysis since less than four studies reported them. The outcome of these risk factors could therefore only be described qualitatively and should be interpreted carefully.

### Interpretation of results

We expected to find that CRP and WBC count were related to complicated diverticulitis. A higher degree of inflammation generally correlates to higher levels of inflammatory parameters. Determining the optimal threshold for use in clinical practice, however, remains a point of debate. Several studies calculated the optimal threshold for CRP level and found cutoff values ranging from 50 to > 175 mg/L [15, 18, 19]. Optimal cutoff values for WBC count were not reported. Generally, WBC levels were higher among patients with complicated diverticulitis (10.4 × 10^9^/L (range 8.7–12.0 × 10^9^/L) versus 14.4 × 10^9^/L (range 12.5–16.3 × 10^9^/L) [18, 19].

As expected, patients with complicated diverticulitis present with more severe symptoms than patients with uncomplicated diverticulitis (diffuse versus localized abdominal pain). This could be accounted for by the presence of diffuse peritonitis [14, 18, 19].

For the other study parameters (comorbidity, DM, BMI, history of previous attacks, steroid use), very little evidence was found. Primary episode, comorbidity, DM, and steroid use could be predictors of complicated diverticulitis [9, 16, 19]. BMI did not show a significant difference in the risk for complicated diverticulitis [14]. In general, patients with (severe) comorbidities are more prone to complications since the underlying illnesses (cardiovascular, pulmonary) could lead to impaired tissue oxygenation and perfusion, which again could lead to an increased risk of perforation. Therefore, the physician should be more vigilant of complicated diverticulitis in these patients. Moreover, the use of steroids abolishes the value of CRP level since steroids suppress the immune system in the inflammatory response. Physicians should take this into consideration when evaluating a patient [16].

### Comparison with other studies

Recently, a systematic review of predictors of acute diverticulitis severity was published. Tan et al. concluded that comorbidity, steroid usage, first presentations, and CRP level of > 175 mg/L are predictive of a more severe disease process with higher likelihood for complications and resultant prolonged clinical course [21]. This is similar to our findings, although we found different cutoff points for CRP level (90, 149.5, and 175 mg/L). A major difference between Tan’s review and the present review is the fact that we used one definition for complicated diverticulitis according the Hinchey classification or Ambrosetti criteria. Tan et al. employed multiple definitions such as risk of surgery, risk of medical treatment failure, and length of hospital stay. This might cause considerable heterogeneity. For this reason and the fact that the Hinchey and Ambrosetti criteria are commonly used in clinical practice, we chose to define our outcome according to these classification systems.

### Implication for clinical practice

In the past decade, our understanding of diverticulitis has increased. The majority of patients have uncomplicated diverticulitis and recover without the use of antibiotics or dietary restrictions. Some studies even suggest treating these uncomplicated cases in a primary care setting [4–6]. A prognostic model would facilitate this vision and may ultimately lead to lower health-care costs for a currently costly disease with high incidence. No studies have previously attempted to develop such a model. This review aimed to identify risk factors for complicated diverticulitis, as a component of such a prognostic model. In our opinion, these risk factors individually have little discriminative value for truly estimating the risk of developing complicated diverticulitis. Combining these risk factors in a full prognostic model might be the next step to aid the treating physician in predicting the course of diverticulitis and setting the right treatment at initial presentation.

We propose a prognostic model in which the following parameters will be included: first episode of diverticulitis, vomiting, constipation, diffuse abdominal pain, rebound tenderness, défense musculaire (as a clinical sign of peritonitis), comorbidity (ASA > I), steroid usage, and CRP level (see Table 2). We chose these parameters based on the results of this systematic review and their applicability in clinical practice for primary care physicians. The weight of each parameter is based on the odds ratios, relative risks, sensitivity, specificity, negative predictive value (NPV), and positive predictive value (PPV) as reported by the included studies. We aimed for the highest sensitivity and NPV since the model should safely rule out complicated diverticulitis in patients suspected of acute diverticulitis. Next, we will carry out a study in a diverticulitis patient cohort to construct a nomogram to calculate the probability of complicated diverticulitis in patients with a suspicion of acute diverticulitis based on the selected parameters. By constructing a nomogram, we can evaluate and support our proposal for a clinical scoring system to make it applicable in clinical practice.

**Table 2:** Risk for complicated diverticulitis

Parameter	Score
Anamnesis	
Pain	
− Localized abdominal pain	0
− Generalized abdominal pain	2
Constipation	2
Vomiting	2
Steroid usage	1
Patient history	
First episode of diverticulitis	2
ASA ≥ II	1
ASA ≥ III	2
Physical examination	
Rebound tenderness	2
Défense musculaire	5
Laboratory parameters	
CRP mg/L	
− 0–50	0
− 50–150	2
− > 150	4

Patients with total score ≥ 5 points should be referred to secondary care

Total score: 0–4 low risk of complicated diverticulitis, 5–8 medium risk for complicated diverticulitis, > 8 high risk for complicated diverticulitis

## Conclusion

Although high-level evidence is lacking, this study demonstrates that CRP level, WBC count, clinical signs (generalized abdominal pain, constipation, vomiting), steroid use, number of episodes, and comorbidity are risk factors for complicated diverticulitis. Individually, these risk factors have little value for truly estimating the risk of developing complicated diverticulitis. Combining them in a prognostic model as proposed by the authors might be the next step to aid the physician in predicting the course of diverticulitis and setting the right treatment at initial presentation.

## Appendix 1

**Table 3:** Search terms

Search terms		
Domain	Determinant	Outcome
diverticulitis	Age Young Old Sex gender Women Men Man Woman Female Male Race Ethnicity CRP “C reactive protein” Leucocytes Leucocyte Leucocytosis Fever Temperature Sodium Hyponatremia hyponatraemia History Pain “Acute abdomen” “Clinical picture” “Clinical feature” “Clinical symptoms” BMI “Body Mass Index” Obesity Obese ASA “American Society of Anaesthesiologists” Co-morbidity comorbidity “Associated disease” “Charlson score” NSAID “Nonsteroidal anti-inflammatory drugs” “Non-steroidal anti-inflammatory drugs” Immunosuppression Immunosuppressed “Risk factors” Riskfactors “Risk factor” riskfactor risk “Alarm symptoms” Prognosis prognostic Predictor predictors prediction	Complicated Complication Complications Abscess Fistula Fistulae Stenosis “Hinchey IB” “Hinchey II” “Hinchey III” “Hinchey IV” Peritonitis “Purulent peritonitis” “Faecal peritonitis” Perforation Perforated Sepsis Resection Surgery Surgical Operation Operative Intervention Death Mortality “Poor outcome”

## Appendix 2

**Table 4:** Hinchey classification

Hinchey	Modified Hinchey
	0 Mild clinical diverticulitis
I Pericolic abscess or phlegmone	Ia Colonic wall thickening and/or confined pericolic inflammation
	Ib Confined small pericolic abscess (≤ 5 cm)
II Pelvic, distant intra-abdominal, or retroperitoneal abscess	II Pelvic, distant intra-abdominal, or retroperitoneal abscess
III Generalized purulent peritonitis	III Generalized purulent peritonitis
IV Generalized fecal peritonitis	IV Generalized fecal peritonitis
	Fistula
	Obstruction

Hinchey EJ, Schaal PG, Richards GK. Treatment of perforated diverticular disease of the colon. Adv Surg. 1979;12: 85–109

## Appendix 3

**Table 5:** Ambrosetti criteria

Mild diverticulitis	Severe diverticulitis
Localized wall thickening of the bowel wall (≥ 5 mm)	Bowel wall thickening (≥ 5 mm) and inflammation of pericolic at + one of the following:
Inflammation of pericolic fat	Abscess
	Extraluminal air
	Extraluminal contrast

Ambrosetti P, Becker C, Terrier F. Colonic diverticulitis: impact of imaging on surgical management—a prospective study of 542 patients. Eur Radiol. 2002;12:1145–1149
